# Status and perspective of protein crystallography at the first multi-bend achromat based synchrotron MAX IV

**DOI:** 10.1107/S1600577525002255

**Published:** 2025-04-04

**Authors:** Ana Gonzalez, Tobias Krojer, Jie Nan, Monika Bjelčić, Swati Aggarwal, Ishkan Gorgisyan, Mirko Milas, Mikel Eguiraun, Cecilia Casadei, Manoop Chenchiliyan, Andrius Jurgilaitis, David Kroon, Byungnam Ahn, John Carl Ekström, Oskar Aurelius, Dean Lang, Thomas Ursby, Marjolein M. G. M. Thunnissen

**Affiliations:** a MAX IV Laboratory Lund University Fotongatan 2 Lund224 84 Sweden; Cornell University, USA

**Keywords:** protein crystallography, synchrotrons, beamlines, time-resolved crystallography, drug discovery, BioMAX, MicroMAX, FragMAX, FemtoMAX

## Abstract

The first multi-bend achromat based synchrotron MAX IV operates two protein crystallography beamlines, BioMAX and MicroMAX. BioMAX is designed as a versatile, stable, high-throughput beamline catering for most protein crystallography experiments. MicroMAX is a more ambitious beamline dedicated to serial crystallography including time-resolved experiments. Both beamlines exploit the special characteristics of fourth-generation beamlines provided by the 3 GeV ring of MAX IV. In addition, the fragment-based drug discovery platform, FragMAX, is hosted and, at the FemtoMAX beamline, protein diffraction experiments exploring ultrafast time resolution can be performed. A technical and operational overview of the different beamlines and the platform is given as well as an outlook for protein crystallography embedded in the wider possibilities that MAX IV offers to users in the life sciences.

## Abstract 2

A comprehensive overview of the protein crystallography beamlines BioMAX and MicroMAX is given. This work introduces FragMAX, a platform for early drug discovery as well potential opportunities for protein crystallography at FemtoMAX, a beamline dedicated to ultrafast experiments.

## 1. Introduction

The impact of structural biology over the last 50 years is tremendous. Protein structures inform us on the function of biological molecules and how they interact and drive the processes of life. Macromolecular structures aid drug development and green chemistry efforts. The growth of structural information is most clearly seen through the Protein Data Bank, inaugurated a little over 50 years ago with initially only 7 structures, to become a database containing over 220 000 structures (August 2024). The main method for structure determination of macromolecules has been macromolecular crystallography (MX). Part of the success of MX lies in the use of synchrotron light for diffraction experiments and the development of dedicated beamlines. The level of MX experimental techniques offered at modern synchrotrons is extremely sophisticated. Owing to the advances made in standardization, instrumentation and computing, methods have flourished as high-throughput data collection as well as remote and fully automated data collection unassisted by users have become available. From a technical perspective, this implies that the beamlines must be extremely reliable and that the hardware and software, including control, data-processing pipelines and data-management programs, must be seamlessly integrated.

In recent years, structural biology has seen enormous changes due to the advances made in cryoEM (Chua *et al.*, 2022 ▸) and the breakthrough of structure prediction for proteins using *AlphaFold* (Jumper *et al.*, 2021 ▸). For MX this has meant a shift in experimentation where, for example, large complexes are no longer pursued by crystallography and *de novo* phasing has become less significant while molecular replacement (MR) is now the main method for obtaining the first phase information. For synchrotron beamlines the collection of single and multiple anomalous diffraction data has become less important, but the capability of obtaining anomalous data should be maintained to enable structure solution when MR fails, for example, because of a lack of an appropriate, experimental or AI-generated MR search model, or to identify elements in anomalous difference maps. Furthermore, X-ray fluorescence techniques can be used for metal identification in macromolecules (Handing *et al.*, 2018 ▸).

New opportunities for MX are also arising as a new era of synchrotron facilities using multi-bend achromat (MBA) technologies in their storage ring design has begun (Shin, 2021 ▸). The use of MBAs leads to a significant reduction in emittance of the electron beam resulting in increased brightness and coherence of the X-ray beam. The technology also provides more stable X-ray beams. The higher brightness and stability allow for faster data collection, reducing the time required for experiments. This is particularly advantageous for high-throughput crystallography, where large numbers of samples are analysed, opening the possibility for crystallography to be used as a screening method in drug discovery (Patel *et al.*, 2014 ▸).

Furthermore, the improved brightness of the beamlines makes it possible to employ serial crystallography approaches that were pioneered at X-ray free-electron lasers (Johansson *et al.*, 2017 ▸). This is because the much brighter focused microbeam, combined with advances in detector technology, allows data collection from micrometre-sized crystals. Therefore, more difficult systems that only produce slurries of microcrystals in crystallization experiments, such as membrane proteins, can be addressed using serial crystallography methods where many thousands of microcrystals are sequentially exposed to X-ray beams. Each microcrystal gives rise to up to one diffraction pattern from a random orientation, therefore providing information over a small portion of reciprocal space. To obtain a complete dataset, diffraction intensities from thousands of isomorphous microcrystals are merged. Data can be obtained at room temperature (RT) as radiation damage does not significantly impact the single frames from which full datasets are built. Finally, structural enzymology including time-resolved measurements in the milli- to microsecond time regime is possible using these approaches.

Thus, the improved performance of MX beamlines at fourth-generation synchrotrons opens up new possibilities for research, including studies of smaller and more challenging crystals, time-resolved experiments, and investigations into more complex biological systems especially when integrated with the use of complementary techniques such as cryoEM and small-angle X-ray scattering (SAXS).

MAX IV laboratory is a synchrotron facility located in Lund, Sweden. The facility was inaugurated in 2016, and it currently operates 15 beamlines situated at 2 storage rings (1.5 and 3 GeV) and 1 beamline at a short pulse facility at the end of the linear accelerator that also delivers electrons at full energy to both storage rings (Robert *et al.*, 2023 ▸). The 3 GeV ring is the first storage ring based on MBA technology (Einfeld *et al.*, 2014 ▸; Tavares *et al.*, 2014 ▸) and therefore MAX IV can be seen as the pioneer of the fourth-generation storage ring light sources (Shin, 2021 ▸). Due to the MBA technology, the 3 GeV ring with a 528 m circumference operating with a 400 mA current has achieved a horizontal emittance of 328 pm rad (Robert *et al.*, 2023 ▸). This makes it a superb source for hard X-ray experiments such as protein crystallography.

MAX IV laboratory operates two beamlines dedicated to protein crystallography that aim to fully cover the experimental possibilities of MX by taking advantage of the characteristics of the 3 GeV ring: BioMAX is primarily dedicated to fully automated high-throughput macromolecular diffraction and it also includes capabilities for MAD and single-wavelength anomalous diffraction, while MicroMAX is mainly focused on serial and time-resolved crystallography. BioMAX was the first beamline to become available to users at MAX IV in 2017 while MicroMAX is the latest, becoming operational in 2024. The two beamlines were designed to be complementary to each other while maintaining some overlap in experimental capabilities. The beamlines share some common instruments, beamline and experiment control software, computing facilities, preparation laboratories, support staff, and operation procedures. Both beamlines benefit from developments in the common areas, while also concentrating on advancing the specific capabilities of each beamline itself. BioMAX hosts the fragment-screening platform, FragMAX, for drug discovery purposes.

In addition, at the short pulse facility located at the end of the linear accelerator, the FemtoMAX beamline can be used to study ultrafast structural dynamics including protein dynamics.

## 2. Technical description of BioMAX

The main BioMAX concept is a highly reliable and stable state-of-the-art MX beamline dedicated to high-throughput experiments but with basic capabilities to cover almost any MX technique (Thunnissen *et al.*, 2013 ▸; Ursby *et al.*, 2020 ▸).

The X-ray source for BioMAX is an in-vacuum, RT, permanent magnet undulator (Hitachi Metals, Japan) with a magnetic period of 18 mm, 111 periods, a minimum gap of 4.2 mm and a maximum *K* value (*K*_max_) of 2.19. The instrument produces a high-brilliance X-ray beam, and it allows tapering of its gap giving wider undulator peaks (Tarawneh *et al.*, 2019 ▸). All four motors controlling the undulator gap are equipped with encoders for precise and fast gap control.

The BioMAX X-ray system consists of an Si(111) horizontal double-crystal monochromator (DCM) (Kristiansen *et al.*, 2016 ▸, FMB Oxford, UK) followed by a Kirkpatrick–Baez (KB) focusing set of mirrors (Ursby *et al.*, 2020 ▸). This combination allows stable operation of the beamline between 6 and 24 keV with a relative bandwidth of 2 × 10^−4^. Four major focusing modes are offered for general user operations: 100 × 100, 50 × 50, 20 × 20 and 20 × 5 µm^2^. The 50 × 50 µm^2^ option is predominantly used for experiments, as it closely aligns with the average crystal size typically employed by users. Continuous fast energy scanning is available (Gorgisyan *et al.*, 2023 ▸) that can be used to measure the X-ray absorption spectrum in the region near the absorption edge of interest in about 1 s.

A beam conditioning unit (BCU) (Cianci *et al.*, 2017 ▸) including two X-ray beam position monitors, three filter wheels for adjustment of beam transmission and a fast shutter is used for beam manipulations and diagnostics. The downstream beam monitor continuously feeds back the beam position to a PID loop that adjusts the KB mirrors pitch to drive the beam to the centre. This system prevents beam drift and ensures changes in the beam focus and energy are totally automated and transparent to the user.

BioMAX is equipped with an ISARA robotics sample changer (IRELEC, France) with a Staubli TX-60. The sample changer has been upgraded and improved since beamline operations began in 2017. Currently, the sample changer has a storage dewar with a capacity for 29 standard universal pucks (Unipucks), giving a total storage of 464 samples. For minimal time loss during exchange of samples, the changer is equipped with a modified double gripper. The modification allows a slightly larger variation in the length of the support pins of SPINE-based sample holders which improves the reliability of the changer. The sample changer can mount *in situ* crystallization plates, and a small plate hotel that can contain up to four plates is mounted on the sample changer table. Additionally, a gripper that can carefully mount samples in quartz capillaries, enabling experiments at RT, is available. Three Unipuck positions are created outside of the storage dewar to contain 48 samples at RT.

Goniometry at BioMAX is performed through an MD3-down micro diffractometer (Arinax, France). This allows fast rotations (up to 800° s^−1^) and raster scanning at 60 Hz. The main configuration includes a mini-kappa goniometer head; a penta-aperture with 100, 50, 20, 10 and 5 µm-diameter apertures that are automatically inserted by the data collection software to match the focus mode by default (although it is possible to overfill the focused beam with a smaller aperture); scatter cleaning capillary tube; scintillator screen for beam visualization; and beam stop. Other goniometer heads, such as a crystallization plate adapter and a high-viscosity extruder (HVE) adapter (Shilova *et al.*, 2020 ▸), are available for *in situ* and HVE-based serial synchrotron crystallography (SSX) experiments, respectively. An Oxford Cryojet supported by a Rapid nozzle exchanger (REX) (Arinax, France) keeps the sample position at 100 K. The REX unit allows for quick exchange with an HCLab humidity controller nozzle.

The area detector at BioMAX was, until spring 2024, an EIGER 16M (Dectris, Switzerland) hybrid pixel detector with a 75 µm pixel size and a 450 µm-thick silicon sensor. To exploit the higher-energy range available at the beamline, the detector was upgraded to a CdTe EIGER2 XE 16M (Dectris, Switzerland). In addition, the EIGER2 XE provides a higher maximum frame rate (560 versus 133 Hz for the EIGER 16M) (Storm *et al.*, 2021 ▸).

## 3. Technical description of MicroMAX

MicroMAX is a beamline conceived and optimized for serial and time-resolved crystallography while also providing high-throughput single-crystal functionality. The high brightness of the beam is well suited for studying small crystals and allows short exposure times. Time-resolved crystallography benefits from using small crystals, regardless of the triggering method, but a small focus and small crystals can also be advantageous for studying structures at RT using serial crystallography, allowing a low-dose exposure for each crystal. MicroMAX has been designed to be flexible in terms of both X-ray beam characteristics and experiment setup possibilities, but to complement BioMAX the focus is on the unique time-resolved possibilities. With this aim the X-ray performance is complemented with, for example, an X-ray microsecond chopper, an integrating detector, and a laser and spectroscopy laboratory.

The X-ray source is an in-vacuum, permanent-magnet undulator (Hitachi Metals, Japan) with a magnetic period of 18 mm, a magnetic lattice length of 2.8 m (156 periods), a minimum gap of 4.2 mm and a *K*_max_ of 2.0. The undulator peaks can be broadened by tapering the undulator device.

To provide the flexibility in beam properties, MicroMAX is built on a choice of different optical elements. For instance, it has two monochromators (both FMB Oxford, UK), a horizontal deflecting Si(111) DCM and a multi-layer monochromator (MLM). The DCM provides energies in the range 5–25 keV with a bandwidth around 2 × 10^−4^ leading to a flux of around 10^13^ photons s^−1^ (at 13 keV in the focused beam, at present FWHM = 14 × 4 µm^2^). The MLM has two different coatings delivering a beam with the more restricted energy range 10–13 keV and a larger bandwidth of 0.4 or 1% depending on the coating which provides an increased flux to more than 10^14^ photons s^−1^ (at 13 keV in the focused beam).

The beam focusing can be achieved using either beryllium compound refractive lenses (CRLs) (Axilon, Germany) only or a combination of CRLs and a set of KB focusing mirrors. The former option is in use since the start of user operations and covers the full energy range 5–25 keV. The second option will be available when the KB mirrors are installed in 2025; while it is restricted to energies between 5 and 20 keV, it will however be able to provide a smaller focal spot of 1 µm diameter – compared with around 10 × 5 µm^2^ for the CRL only option – and yield a somewhat higher flux. The CRLs allow fast changes to the beam focus that is used in combination with beam apertures in the same way as at BioMAX to tailor the beam size to the sample. The KB mirror pair will have fixed curvatures and an in-house developed mechanical system based on a similar instrument developed for the MAX IV nanoprobe beamline NanoMAX (Carbone *et al.*, 2022 ▸). Switching between the two different systems will lead to a change in direction of the X-ray beam resulting in a change in position of the focal spot by 7 mm vertically and 5 mm horizontally.

**Figure 1:**
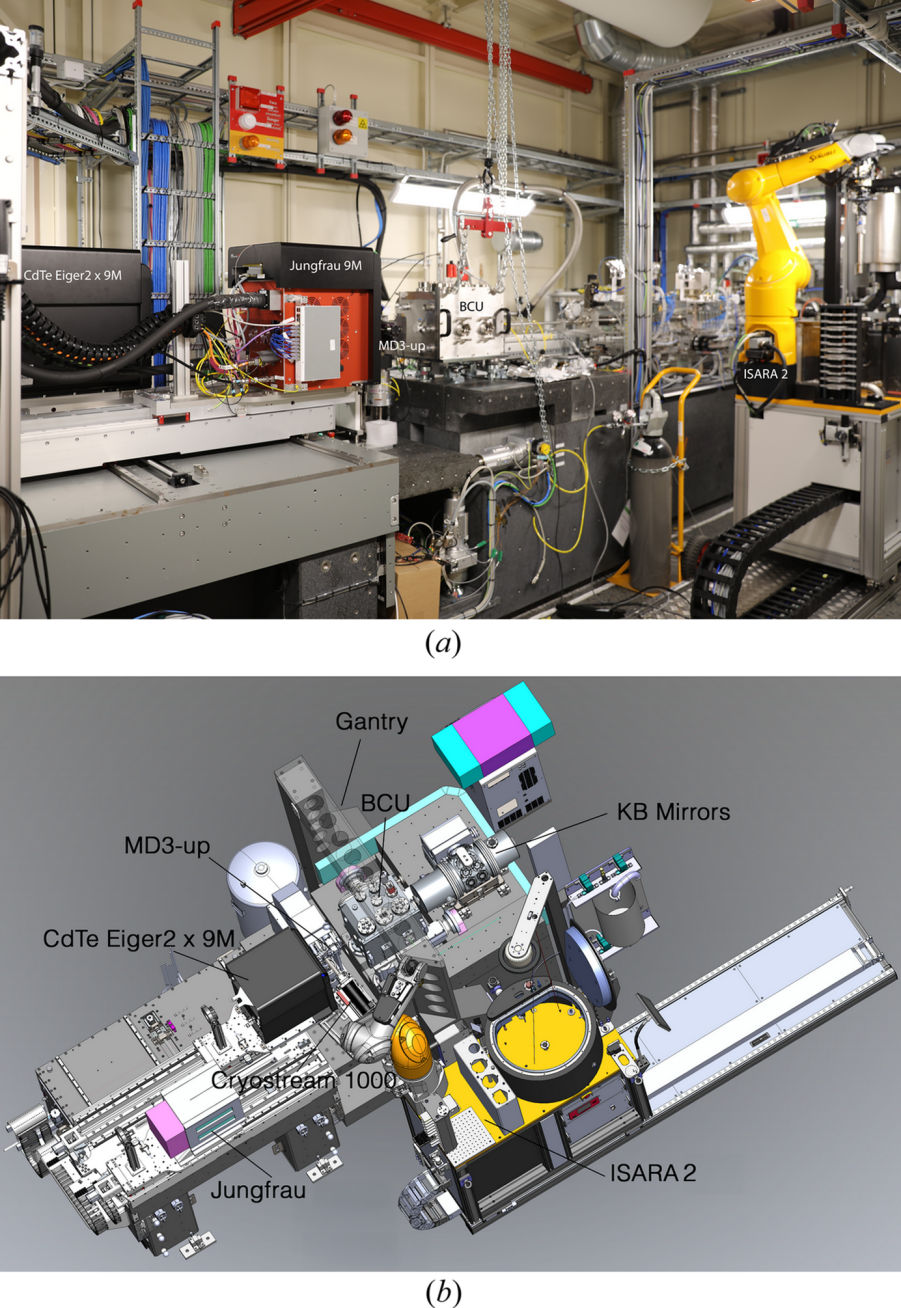
Overview of the EH1 experimental hutch of MicroMAX. (*a*) Photograph of the current setup with different instrumentation noted. (*b*) CAD drawing of final setup at EH1 including the gantry for sample delivery instrumentation installation.

To deliver high experimental flexibility, MicroMAX has two experimental hutches (EH1 and EH2). EH1 hosts the primary endstation based on an MD3-up diffractometer (Arinax, France) on an in-house developed sample table, an ISARA2 (Irelec, France) sample changer and a CdTe EIGER2 X 9M detector as well as a JUNGFRAU 9M (PSI, on loan), both mounted on an in-house developed diffraction detector table (Fig. 1 ▸). Cryo-cooling of samples is facilitated by a Cryostream 1000 open-flow cryo-cooler (Oxford Cryosystems, UK).

The MD3-up, like the MD3-down at BioMAX, is an integrated diffractometer system allowing fast rotations and raster scanning. There are five different goniometer heads available at MicroMAX: a single axis and a mini-kappa for SPINE-based sample holders, a fast-scanning fixed-target stage that scan solid supports up to around 30 × 30 mm^2^, a holder for crystallization plates, and an empty head to make less standardized experiments possible and provide space for user developed experimental setups. The in-house developed sample table onto which the MD3-up is mounted can reposition the diffractometer when changing the focusing from CRLs only to the CRLs–KB mirror combination, but is then kept in its aligned position and the beam is steered by the X-ray lenses to compensate for any beam drift.

The storage dewar of the ISARA2 sample changer has the same capacity as at BioMAX, 29 Unipucks, and there is also storage for crystallization plates and SPINE samples at RT that can be mounted using the sample changer. SPINE sample holders are mounted using a double gripper to reduce exchange time and there is also an empty tool that allows custom equipment fitting. The entire ISARA2 system is mounted on a translation system that allows the user to position the equipment upstream of the sample position to create extra space for those experiments where a sample changer is not needed.

The two area detectors at the beamline are mounted on sets of parallel rails on an in-house developed detector table allowing five degrees of freedom. The horizontal translation provides easy exchange between the detectors. The CdTe EIGER2 X has a maximum count rate of 10^7^ photons s^−1^ and a maximum frame rate of 245 Hz. A central zone of interest with a subset of the modules available can be used to collect data at 560 Hz. Kilohertz serial crystallography is enabled through the second integrating pixel detector, a JUNGFRAU 9M (Mozzanica *et al.*, 2018 ▸) on loan from the PSI, Switzerland.

EH1 will be equipped with a gantry to support the sample cryo nozzle and other auxiliary equipment. It will improve the use of the space around the sample and user access to the equipment. It will also serve to readjust positions of the different instruments upon change of the optical pathway.

A BCU was installed in autumn 2024. It has similar functionality as the BCU at BioMAX, but it will also include an X-ray chopper (Celeroton, Switzerland) providing pulses down to 10 µs with variable repetition rates. A timing system based on a PandaBox (Zhang *et al.*, 2017 ▸) is used for synchronization of the different components such as the X-ray chopper, shutters, detectors, laser, diffractometer/sample delivery systems and X-ray beam diagnostics.

In EH2 a laser laboratory and an offline transient absorption spectroscopy laboratory are available to obtain spectroscopic information over similar time scales to the diffraction experiments. The laser system consists of a class 4 NT230 nanosecond tuneable laser (Ekspla, Lithuania) which, through optical fibre guiding, can serve as the pump source for pump–probe crystallographic experiments in EH1 and spectroscopic measurements on photosensitive systems.

## 4. Experimental control, data handling and evaluation

For low-level instrumentation control, MAX IV Laboratory uses *Tango* (https://www.tango-controls.org/) and *Sardana* (https://www.sardana-controls.org/) (Coutinho *et al.*, 2011 ▸). This software is supported by the MAX IV technical division. Both BioMAX and MicroMAX employ *MXCuBE* as the user interface and higher-level beamline control software. The *MXCuBE* (*Macromolecular Xtallography Customized Beamline Environment*) project started at the European Synchrotron Radiation Facility (ESRF) (Gabadinho *et al.*, 2010 ▸) and was adopted by several other European MX beamlines as their main beamline-control software. Current development of *MXCuBE* is performed through a consortium supported by most European synchrotrons as well as several facilities elsewhere (https://www.mxcube.org/, Mueller *et al.*, 2017 ▸). *MXCuBE3* was developed by the ESRF and MAX IV (Oscarsson *et al.*, 2019 ▸) as a web-application and it runs in any recent browser. It was first employed at BioMAX when that became operational in 2017 (Mueller *et al.*, 2017 ▸). *MXCuBE4*, a recent upgrade based on Python 3, is used at MicroMAX and will be deployed for users at BioMAX in 2025.

Experiments and tasks available to MAX IV users through *MXCuBE* include interactive dose estimation, automatic X-ray centering supported by on-the-fly analysis by *Dozor* (Melnikov *et al.*, 2018 ▸), auto-loop centering (in-house development), standard rotational data collection, interleaved and inverse beam multiple-wavelength anomalous diffraction (MAD) data collection, injector-based crystallography experiments including time-resolved SSX as well as fixed-target scanning SSX.

In addition, *MXCuBE3* and *MXCuBE4* provide access to some scripts to perform complex procedures involving multiple beamline systems, such as restoring the X-ray beam after an electron beam loss in the storage ring, alignment of the MD3 apertures, flux calculation and sample annealing, among others.

Additional scripted operations available to staff at BioMAX include a shell script that automatically prepares the beamline for user operation. This script checks the status of major devices, resets beamline servers, aligns the X-ray beam, calculates the flux, collects an air scatter image to check for correct alignment of MD3 components, measures the direct beam position for SSX experiments and cleans up open processes. All steps are logged, and emails are generated to staff on duty with a concise summary. A similar script will be implemented at MicroMAX once the operation startup procedure is fully determined and tested.

The seamless integration of beamline equipment with data analysis supported by *MXCuBE* has made it possible to implement fully automated rotation data collection at BioMAX. The samples are mounted on the goniometer by the sample changer, automatically aligned using optical centering of the loop followed by an X-ray scan in two directions and data collection according to the instructions provided by the users in the sample list. Implementation of the sophisticated automated data collection workflow developed by Global Phasing Limited that includes sample characterization is also planned.

The EIGER2 detector output at both beamlines is generated and collected using the *Eiger2 Filewriter* and *Stream* interfaces (Burian *et al.*, 2023 ▸).

After rotation data collection, the program *Dozor* (Melnikov *et al.*, 2018 ▸) performs a peak search on each image; the plot of the number of detected spots, a diffraction quality score and the apparent resolution limit are available a few seconds after the datafiles are written to disk. Data-processing pipelines for rotation datasets such as *fast_dp* (Winter & McAuley, 2011 ▸), *Ednaproc* (Incardona *et al.*, 2009 ▸) and *autoproc* (Vonrhein *et al.*, 2011 ▸) are used for indexing, processing and data reduction, respectively. A modified version of *fast_ep* (Winter & McAuley, 2011 ▸) (https://github.com/DiamondLightSource/fast_ep) is triggered for automated experimental phasing if a significant anomalous signal is identified in any data-processing pipeline. There are advanced plans to deploy an in-house developed MR pipeline that allows users to either upload a PDB file directly as a search model or supply an input amino acid sequence or UniProt ID from which *AlphaFold* (Jumper *et al.*, 2021 ▸) is used to generate search models. These models are prepared and analysed using tools in *Phenix* (Liebschner *et al.*, 2019 ▸); *Dimple* from the *CCP4* package (Agirre *et al.*, 2023 ▸) is used for the MR analysis.

**Figure 2:**
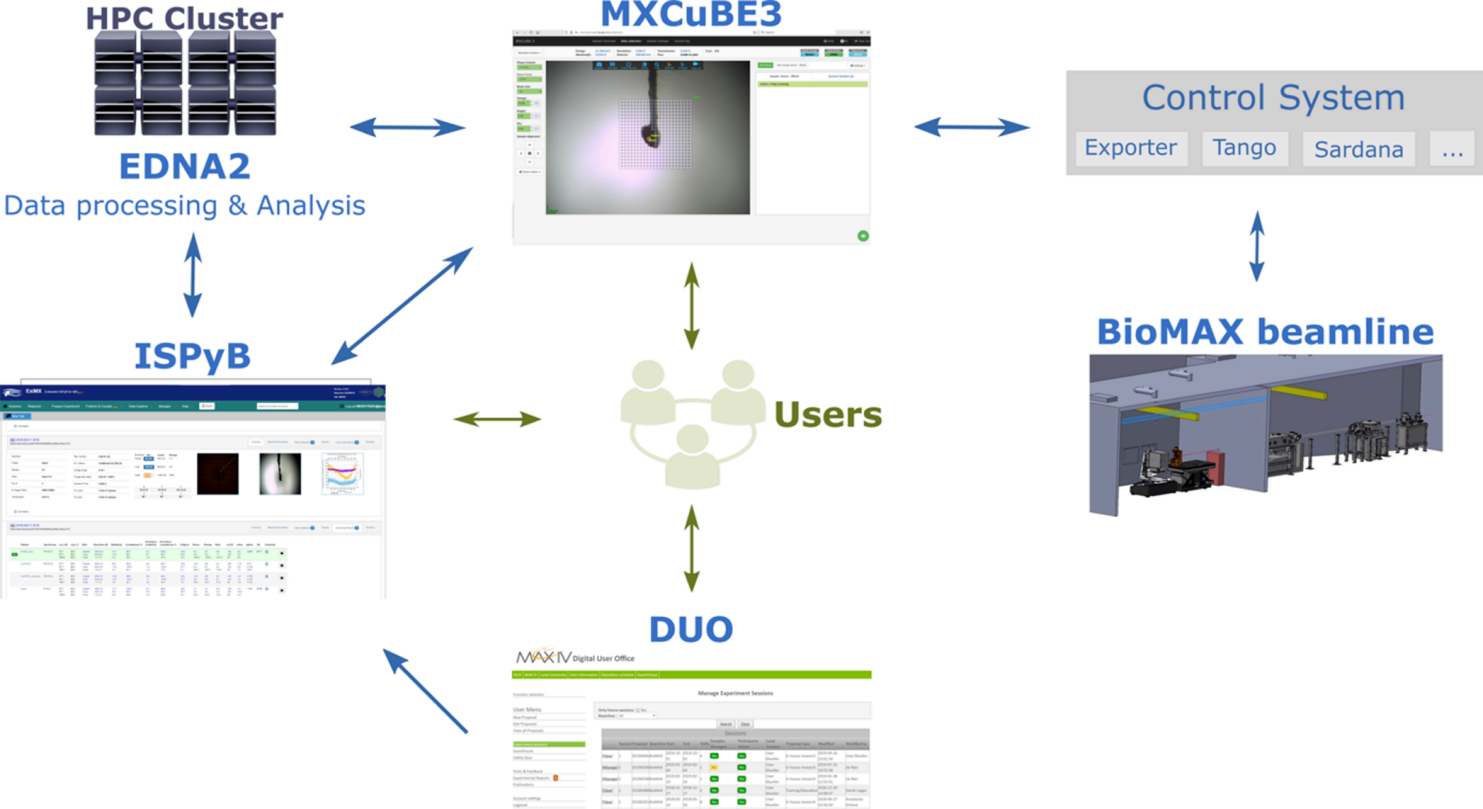
Overview of control and analysis software at BioMAX and MicroMAX. Arrows in green show the interactions between users and software, while arrows in blue show the communication between different software.

All the above described data processing, reduction and phasing pipelines are managed in *EDNA2* which interacts both with *MXCuBE3* and the laboratory information management system (LIMS) *ISPyB* (Delagenière *et al.*, 2011 ▸). EDNA was originally designed as a framework for different plug-in-based applications for a variety of X-ray-based experiments at synchrotrons (Incardona *et al.*, 2009 ▸) for online analysis purposes. The MX part of *EDNA* is presently supported through the *MXCuBE* consortium and *EDNA2* is a complete rewrite of the original *EDNA MX* package based on Python 3 (https://github.com/olofsvensson/edna2). An overview of the different analysis and control software is shown in Fig. 2 ▸.

The EXI interface to *ISPyB* (Santoni, 2019 ▸) is used for sample tracking as well as results and metadata logging. *ISPyB* has been, like *MXCuBE*, developed through a consortium of different synchrotron facilities. Application programming interfaces are available through which other LIMS systems such as *Icebear* (Daniel *et al.*, 2021 ▸) can access the underlying database. MAX IV is currently collaborating in the development of a new *ISPyB* database and interface rewritten in Python.

**Figure 3:**
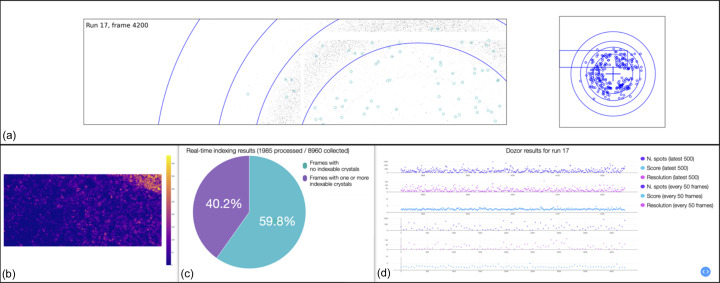
Screenshot of real-time data processing results at MicroMAX, as viewed from the beamline control computers. (*a*) Real-time view of diffraction patterns superposed with identified spot positions. The panel on the right shows the entire detector frame with resolution rings marking 4.0, 3.0, 2.5 and 2.0 Å. The rectangle in blue corresponds to the zoomed-in region displayed on the left panel. (*b*) View of the diffraction quality score calculated in *Dozor* over the two-dimensional grid for a 2D-raster data collection mode. (*c*) Pie plot showing the results of the real-time estimate of the indexing rate in *CrystFEL*. The fraction of frames containing one or more indexable crystals is shown in purple and the fraction with no indexable crystals is shown in blue. (*d*) Real-time spotfinding results from *Dozor*. The top three plots show the number of detected peaks, the diffraction quality score and the apparent resolution limit as a function of the frame number for the last 500 frames of the current detector run. The bottom three plots show the same indicators for the whole of the current detector run, sampling 1 of 50 frames.

For SSX experiments, on-the-fly feedback about data quality is critical and an in-house real-time data analysis system is under development. It consists of a highly parallel program running on multiple dedicated processing nodes. Data and metadata are received through the *Eiger2* stream interface. Independent workers carry out simultaneous *Dozor* peak searches and the results are plotted and displayed during data collection. The detector coordinates of detected spots are fed into a queueing system and used to determine a real-time estimate of the indexing rate from a subset of frames using *indexamajig* [*CrystFEL* (White *et al.*, 2012 ▸)]. Fig. 3 ▸ shows a view of the real-time analysis results. Spot positions are stored in HDF5 format together with symbolic links to the raw data for further processing. *Indexamajig* input files, including a detector geometry file and, if cell information is present in the metadata, a unit-cell file, are generated for each data collection, allowing indexing and integration of all frames as soon as a data acquisition is complete. Integration of SSX data processing results in *ISPyB* is ongoing.

The computing infrastructure for the MX beamlines is organized through a blend of dedicated computers at the beamline and a high-performance cluster (HPC) that serves all beamlines at MAX IV. However, 784 cores from the 2560 cores and 48 GPUs available at the MAX IV HPC are currently reserved for BioMAX, guaranteeing the fast experimental feedback users require during experiments. Currently an additional 960 cores, fully dedicated to MicroMAX, are being added to the cluster.

The experimental beamline control is run from a clustered virtual machine environment. The data acquisition system consists of a server cluster that receives detector data, applies corrections and stores it permanently. This cluster features high-bandwidth networks for data reception, storage and streaming to an HPC system for real-time analysis. Data storage uses a tiered, clustered system with low-latency networks, starting with a fast tier for collection and processing, then migrating to slower tiers for re-analysis or archiving. Data are backed up to tape, catalogued in *SciCat* and retained for at least seven years. Users can transfer data externally via *Globus Connect*, *sftp* or custom tools, and access archived data through *SciCat*.

MAX IV users have 24/7 remote access to their data after the experiment. Autoprocessing results can be browsed and downloaded via the *EXI* web interface. Additionally, the users can log in to the HPC for manual data reprocessing. *PReSTO*, a software stack for integrated structural biology developed for the National Academic Infrastructure for Supercomputing in Sweden, provides a suitable environment to run the most used MX packages, ensuring efficient use of the HPC computing resources.

## 5. FemtoMAX

The multi-experimental beamline FemtoMAX is not, in contrast to BioMAX and MicroMAX, based at the 3 GeV storage ring but at the short pulse facility located after the long LINAC that feeds the two storage rings at MAX IV. The beamline, operational since 2021, is dedicated to time-resolved experiments in the femtosecond to picosecond regime (Enquist *et al.*, 2018 ▸). It is part of the MAX IV portfolio, with beam time proposals for FemtoMAX managed through the MAX IV user office as part of the regular beam time calls. FemtoMAX is the only beamline at MAX IV that utilizes the MAX IV linear accelerator as an electron source to produce photon bursts <100 fs long. FemtoMAX thus combines the stability of a storage ring with the temporal resolution of an FEL. The beamline is equipped with multiple sample environments and optics serving a broad user community. The X-ray source for FemtoMAX is composed of two 5 m long in-vacuum undulators (Hitachi Metals, Japan) with a phase shifter placed in between the undulators. Thus, a 666-period short-period undulator with a 10 m active length was created to cover the energy range 1.8 – 20 keV.

The optical system for FemtoMAX offers alternatives. Two focusing options as well as two different monochromators are installed.

For focusing, a toroidal Rh-coated Si mirror (FMB Oxford) with an incidence angle of 0.14–0.18° is installed which can focus the full X-ray energy range at FemtoMAX to a focal spot of ∼40 × 80 µm. For experiments where a smaller focal spot is required, a set of Be lenses is installed to obtain a focal spot FWHM diameter of <15 µm. This option limits the energy range (5–16 keV) somewhat and reduces the X-ray beam intensity. Two monochromators are installed at FemtoMAX. A DCM (FMB Oxford) houses two sets of crystals [InSb(111), Si(111)] that can be interchanged by a translation stage. InSb (111) extends the energy spectrum to softer X-rays and provides a wider bandwidth compared with Si(111). A MLM (Rigaku Innovative Technologies) is required to obtain the highest possible flux for wide-angle X-ray scattering experiments on liquids while at the same time suppressing the low-energy tail in the undulator spectrum. Three different multi-layer mirror pairs have been coated onto the same substrates together with the harmonic rejection stripe to conveniently optimize the performance for different wavelength ranges. The resulting flux is 6 × 10^6^ photons per pulse at 8 keV.

Like the optics, the sample environment at FemtoMAX is flexible and offers alternatives with two different endstations currently installed (an in-vacuum and an in-air endstation) and five different 2D detectors available for data acquisition. In addition, users can bring their own experimental setup for incorporation into the beamline. For protein crystallography experiments, the in-air endstation is used which consists of a Kappa Goniometer TS-48084 and a 7-axis tilt platform from HUBER Diffraktionstechnik GmbH and Co. The main detector for MX experiments is an adapted PILATUS3 1.2 M (Dectris, Switzerland). The mode of operation of this detector is time over threshold and it adapts the photon-counting detector into a charge integrating device while maintaining the benefit of extremely low noise (Enquist *et al.*, 2018 ▸). Using this detector, Jensen *et al.* (2021 ▸) were able to collect diffraction data on bovine trypsin with a comparable quality to the data obtained at BioMAX.

The beamline has a laser-laboratory located on the floor above the beamline X-ray hutch to provide the beamline with a source for pump–probe experiments. The main laser is a cryo-cooled kHz Ti:sapphire amplifier (KM Laboratories Red Wyvern). It can run at a repetition rate up to 1 kHz and delivers pulses with a duration of 50 fs and pulse energy of 11 mJ at a central wavelength of 800 nm. The laser system also includes an optical parametric amplifier (OPA; TOPAS HE from Light Conversion) with mixing stages to cover the wavelength range 0.2–10 µm. Out of the available 800 nm laser power, 10% is split off and used for diagnostics whereas the remaining 10 mJ pumps the OPA or can be used for experiments.

In addition to ultrashort laser excitation, FemtoMAX also provides laser-generated terahertz pulses as a pump source. The terahertz radiation is generated through optical rectification in the organic crystals DAST and DSTMS (Vicario *et al.*, 2015 ▸; Hauri *et al.*, 2011 ▸). These crystals have the tremendous advantage that, with a pump wavelength of 1500 nm, the group velocity of the optical pulse matches the phase velocity of the generated terahertz radiation in a collinear geometry. The simple phase-matching scheme vouches for an easy setup and alignment as well as a high conversion efficiency (∼1%). Both these types of crystals generate a broad output spectrum centred at approximately 2.1 THz. With the available pump energy the converted pulse energy is typically 10 µJ, which, focused down to a spot size of ∼200 µm, gives a peak electric field of a few megavolts per centimetre. Due to high absorption in air, experimental setups involving terahertz pump pulses are preferably built to be very compact (*i.e.* with as short as possible in-air transport) or alternatively in a dry or vacuum environment.

The experimental control system at FemtoMAX for data collection uses the *Sardana Framework* (Coutinho *et al.*, 2011 ▸). FemtoMAX uses custom made scans to synchronize the detectors and laser delays with the X-ray bursts incoming from the linear accelerator. The control system is standardized and uses standard Python commands and scripting. Additionally, custom macros can be created by the beamline staff and users to fit specific experimental needs. Standard online data analysis tools such radial integration or the sum of the region of interest have been implemented and are available for the users.

**Figure 4:**
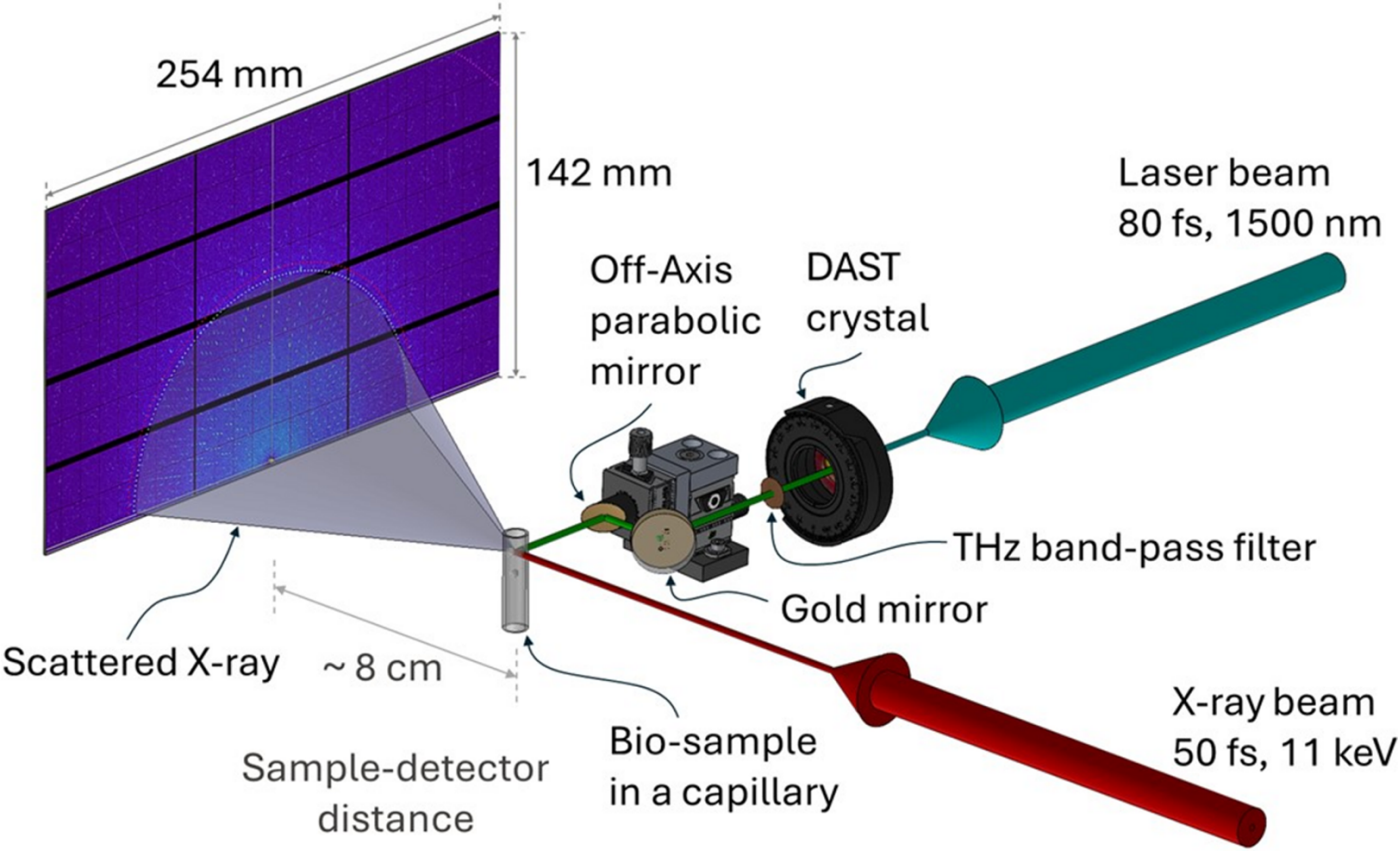
Experimental setup at FemtoMAX. A direct X-ray beam path with scattered beams from a biosample in a capillary is depicted in red. The cyan line to the DAST crystal indicates the beam path of pump laser pulses, and the green line after the DAST crystal shows the generated terahertz beam path. The terahertz beams are steered and focused on the biosample by an off-axis parabolic gold mirror. The X-ray beam incident to the sample diffracts, and the diffracted rings are captured by the 2D area detector. The 254 × 142 mm^2^ detector area contains approximately 1.2 million pixels, each 172 × 172 µm^2^ in size, divided into 12 modules.

To further understand protein dynamics, visualize collective movements in protein crystals and model them in terms of coherent waves, ultrafast experiments were conducted to observe terahertz-induced deformations in bovine trypsin crystals at FemtoMAX (Gagnér *et al.*, 2024 ▸) (Fig. 4 ▸). Although no evidence of terahertz-induced unit-cell perturbations was found, a strong correlation was observed between the direction and magnitude of atomic displacements and specific atom types, with only a weak correlation between atom displacements and their locations. The studies show that long ultrafast experiments probing protein crystals with a low intensity beam using terahertz perturbations as provided by FemtoMAX can yield new information on protein dynamics.

## 6. FragMAX

The peak throughput capacity at BioMAX of almost 500 datasets per day inspired the development of the FragMAX platform for crystal-based fragment screening to aid in early drug discovery.

The FragMAX facility is a platform for crystallographic fragment and ligand screening (Lima *et al.*, 2020 ▸; Kanchugal *et al.*, 2025 ▸). Established in 2020, the facility has undergone significant changes since then (manuscript in revision). The platform consists of three primary elements: (i) a high-throughput crystal preparation laboratory, (ii) automated diffraction data collection at the BioMAX beamline and (iii) software tools for large-scale data processing. The crystal preparation laboratory is currently located within the BioMAX sample preparation laboratory. The laboratory employs highly automated workflows utilizing robust and straightforward liquid-handling robotics for crystal soaking. Specifically, an Opentrons liquid handling robot is used for cherry picking and soak plate preparation, followed by a programmable 96-channel pipette for rapid crystal soaking. Furthermore, crystal mounting and sample logistics are simplified with the use of a Crystal Shifter (Wright *et al.*, 2021 ▸) and EasyAccess frame (Barthel *et al.*, 2021 ▸).

All instruments in the laboratory are supported by a custom-made project management software (*FragMAXdb*) and a database, ensuring comprehensive data capture from protein batch to PDB deposition. The current implementation allows for the preparation and subsequent data collection of 200–300 crystals per day, and its low-cost equipment lends itself to further parallelization with minimal investment. Further, FragMAX has access to a wide range of equipment for protein crystallization and crystal optimization through its collaboration with the Lund Protein Production Platform (LP3). The facility offers access to several fragment libraries and users can choose what fits their needs best. FragMAX currently offers four main collections: the in-house developed FragMAXlib library (158 fragments) (Lima *et al.*, 2020 ▸), the EU-Openscreen library (968 fragments) (Jalencas *et al.*, 2024 ▸), the DSI-poised library (860 fragments) (Cox *et al.*, 2016 ▸) and the MiniFrags library (80 fragments) (O’Reilly *et al.*, 2019 ▸). Importantly, while FragMAX was designed as a fragment-screening platform, its workflows are generic and can support any large-scale protein–ligand study, allowing users to bring their own compound collections and screening sets.

X-ray diffraction data collection is conducted at the BioMAX beamline using cryo-cooled samples. Since January 2023, all crystals prepared at FragMAX have been collected in automated mode at BioMAX. This decouples crystal preparation from data collection and enables efficient use of the available beam time at BioMAX. Though users are typically not involved in data collection, careful diffraction tests are conducted by staff and discussed with users to ensure optimal results before embarking on large-scale screening. The implementation of fully automated data collection at BioMAX was essential for increasing productivity at FragMAX.

**Figure 5:**
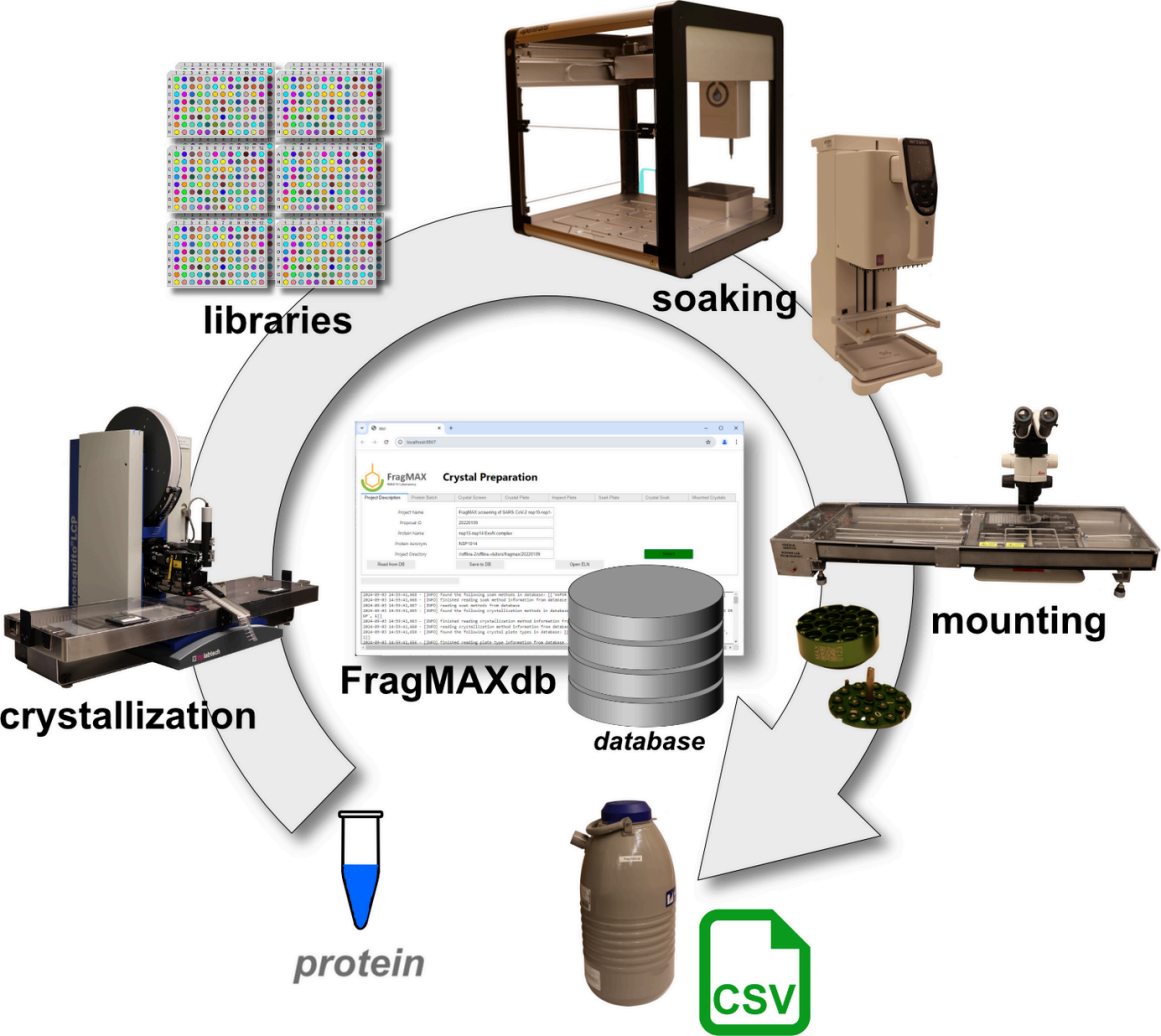
Schematic of the FragMAX crystal preparation workflow. The workflow illustrates the key stages of crystal preparation for fragment screening at FragMAX. Users provide protein samples, which are crystallized by the facility. Crystals are prepared for fragment screening using an optimized and streamlined process. The workflow is managed via *FragMAXdb*, a dedicated project management software. All associated metadata are systematically recorded in a project database to support reproducibility and data integrity.

Finally, FragMAX utilizes results from the automated data-processing routines implemented at BioMAX. The platform has developed two data analysis platforms for rapid structure solution and hit finding. *FragMAXapp* is a web application for interactive data analysis (Lima *et al.*, 2021 ▸), whereas *FragMAXproc* is a command-line-based pipeline integrated with the project database. Both tools enable rapid initial structure refinement, ligand-restraint generation and hit finding using the *PanDDA* algorithm (Pearce *et al.*, 2017 ▸). FragMAX takes full advantage of the MAX IV HPC, ensuring that users receive a full set of results shortly after data collection, which they can transfer to their home laboratories for further analysis. A schematic of the full workflow of FragMAX is given in Fig. 5 ▸.

Since its establishment, FragMAX has successfully conducted 26 crystallographic screening campaigns, serving both academic and industrial clients. Screening campaigns have ranged from 100 to 1800 individually prepared crystals, with hit rates varying from 1 to 10%, depending on the target protein and crystal form. Its workflows have been effectively applied to projects ranging from initial fragment screening to iterative compound optimization. User feedback has been instrumental in continuously refining facility workflows and automation processes, enhancing the overall user experience.

## 7. Operations and experiments

Since BioMAX started operations in 2017, the beamline has contributed with over 900 entries to the Protein Data Bank (PDB, https://biosync.rcsb.org/) and data from it have been the basis for over 200 publications (https://publications.maxiv.lu.se/BioMAX).

**Figure 6:**
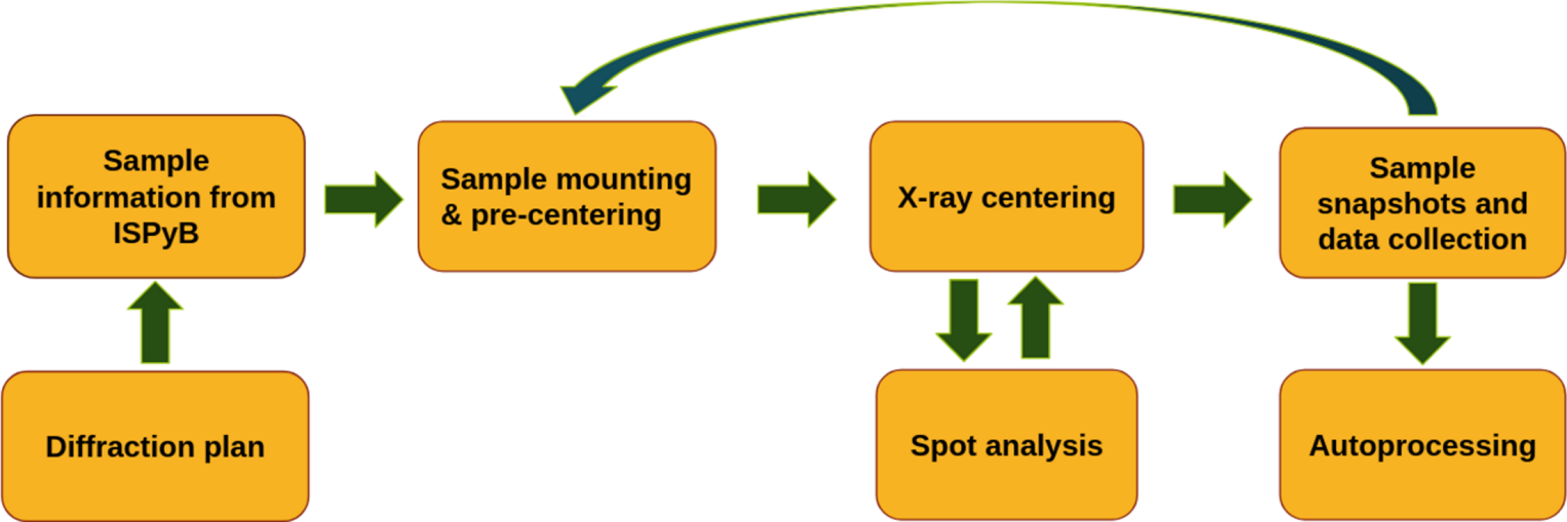
Scheme of the unattended data collection currently implemented at BioMAX. Collection of sample snapshots is optional. Except for the automated X-ray centering, the procedure relies on routines previously available in *MXCuBE*.

The most common experimental setup requested by users at BioMAX is for single axis data collection at cryogenic temperatures through remote access. These data are collected by default using 0.1° rotation and 2 ms exposures per image with an X-ray beam transmission between 50 and 100% depending on the sample. Efforts to increase the reliability of the beam alignment, the sample changer operation, and the gradual increase of the throughput and introduction of automated procedures culminated in fully automated data collection (Fig. 6 ▸). In September 2024, the Industrial Relations Office and the MX group at MAX IV initiated a mail-in service for industrial users based on running unattended experiments during unused evening or overnight shifts. So far, demand for this service has been high, with at least one industrial group sending samples almost every week. Over the past years we have seen a clear increase in industrial users, while the number of academic users has oscillated and overall remained more stable. Additionally, the utilization of FragMAX has grown considerably among both academic and industrial users, requiring a significant allocation of beam time. As a result, BioMAX remains fully booked, reflecting a strong and sustained demand for its capabilities.

Control of all the tools required to conduct the experiment from a single interface facilitates remote access to experiments. BioMAX remote users connect to a dedicated virtual machine using a remote desktop client (Cendio ThinLinc) after establishing a secure VPN connection to the beamline network. The remote desktop provides access to *MXCuBE* and image-visualization software while disallowing low-level control software for the beamline instrumentation. Remote access is granted by default to all the user groups during the duration of their beam time and most experiments at cryo-conditions are carried out fully remotely.

A development priority for MX at MAX IV is to extend the capabilities for automation and remote access to other techniques, especially SSX and RT experiments. The purpose of these efforts is to promote RT experiments by making them as accessible and easy for users as data collection at cryo-temperature. RT data collection is important for a better understanding of protein dynamics relevant to function especially as cryo-cooled protein structures show distorted conformational ensembles (Keedy *et al.*, 2014 ▸). RT structures can reveal alternative conformations of side chains and backbones aligning better with solution data and even more relevant to biological function (Woldeyes *et al.*, 2014 ▸).

Ligand-binding studies comparing complex structures solved at room and cryo temperatures show similar differences with alternative binding poses, unobserved binding sites (Skaist Mehlman *et al.*, 2023 ▸) or at different sites (Fisher *et al.*, 2015).

BioMAX currently provides an MD3-compatible crystallization plate adapter that supports several types of plates. Plate navigation, a special crystal centering procedure and *in situ* data collection are available in *MXCuBE3*. Up to 30° of data can be collected from single crystals. An alternative approach is to carry out a mesh scan over the area of the crystal drop, similarly to fixed-target SSX data collection. Further integration with *ISPyB* to support sample annotation is needed. Implementation of pipelines to obtain complete datasets from serial images or partial single-crystal datasets is an important part of these developments.

Development of automated approaches for sample handling and exchange at RT is also an ongoing project. Adaptation of the ISARA sample changer to load samples mounted in capillaries, Mitegen plastic sleeves or SSX grids at RT has been completed. The samples are mounted in standard Unipuck lids, placed outside of the storage dewar. Automated exchange of crystals in capillaries, combined with a robust crystal transport system, opens the door to collecting both neutron and X-ray data on the same crystal and of interest to the European Spallation Source, Europe’s most advanced and high-power neutron spallation source, under construction in Lund (Lindroos *et al.*, 2011 ▸). These improvements will also aid serial approaches for drug discovery to avoid temperature artefacts (Dunge *et al.*, 2024 ▸). Small-molecule experiments, often done at RT, have also benefited from this facility (Li *et al.*, 2023 ▸). The next step in this project is access to the RT tools from *MXCuBE* and implementation of sample management in *ISPyB*.

**Figure 7:**
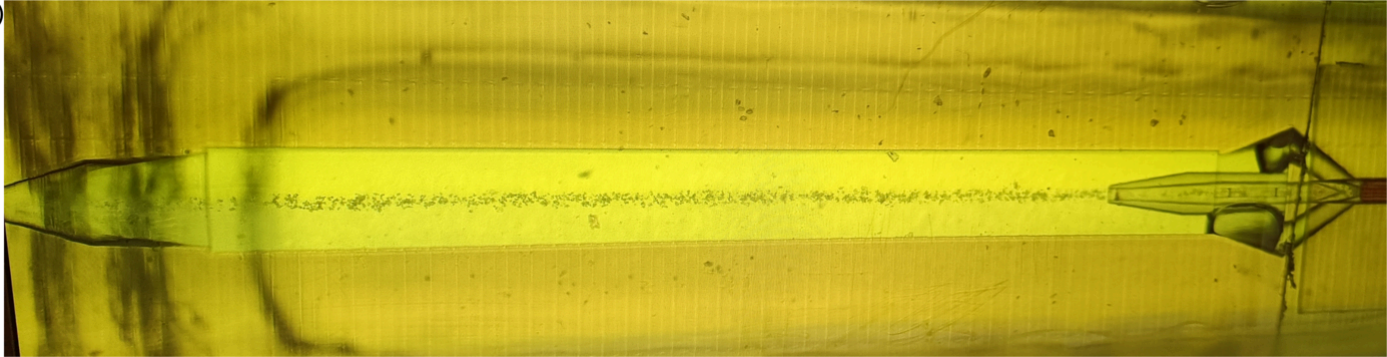
The Adaptocell microfluidic device (Bjelčić, 2023 ▸) available for solution-based serial crystallography experiments. The image shows a focused stream of lysozyme crystals.

Until completion of the MicroMAX construction in 2024, BioMAX was used to test and explore different flow-based and fixed-target delivery systems. For flow-based systems, an adaptor for an HVE injector has been developed for the MD3-down (Shilova *et al.*, 2020 ▸). In collaboration with the group of Professor Neutze the serial-X flow cell was developed which provides more flexibility and easier sample handling during an experiment than an HVE injector (Ghosh *et al.*, 2023 ▸) for samples in high-viscosity media such as LCP-based samples. For solution-based samples, an AdaptoCell microfluidic device (Bjelčić, 2023 ▸) is now available (Fig. 7 ▸). Further development of these systems continues at MicroMAX.

**Figure 8:**
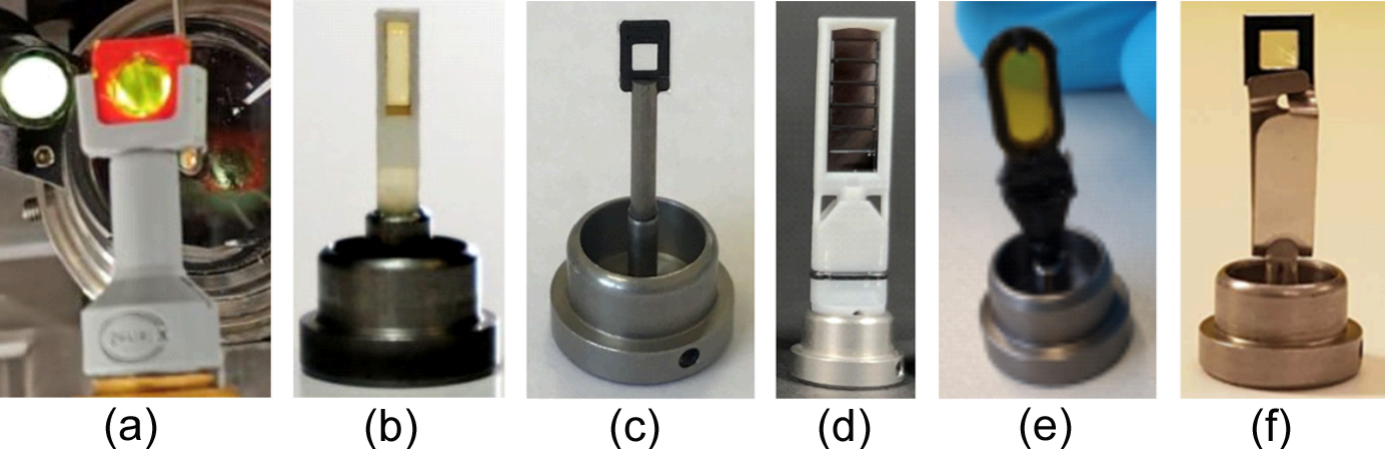
High-degree rotation fixed-target chips for serial crystallography experiments. (*a*) Serial-FiX, chip (Banacore, 2025 ▸). (*b*) MiTeGen (Illava *et al.*, 2021 ▸). (*c*) SwissMX (Karpic *et al.*, 2020 ▸). (*d*) Suna (Lieske *et al.*, 2019 ▸). (*e*) XtalTool (Feiler *et al.*, 2019 ▸). (*f*) Silson (Coquelle *et al.*, 2015 ▸).

Several chip-based solutions for fixed-target serial crystallography are based on SPINE format sample holders and therefore fully compatible with both the MD3-down or the MD3-up (see Fig. 8 ▸ for an overview). Finally, in-house developed technology using silicon nitride chips arranged in a ‘sandwich’ configuration enabled successful data collection on de­oxy and met-hemoglobin samples. The validity of these results was further confirmed by X-ray absorption spectroscopy measurements performed on the same chips at the hard X-ray absorption and emission spectroscopy Balder beamline at MAX IV (Bjelčić *et al.*, 2023 ▸).

Initial tests of the integrating pixel JUNGFRAU detector (Mozzanica *et al.*, 2018 ▸) performance for SSX experiments at MAX IV were carried out at BioMAX, in collaboration with the PSI. The tests involved several injector-based SSX experiments including time-resolved studies using a JUNGFRAU 4M. A pump–probe laser setup was used to study a light-driven sodium pump (KR2). A time range of 130 ms was chosen, including 10 ms dark state, 10 ms excitation by laser and 110 ms probe period, and data were collected at 1 kHz for around 10 h. The data from the 10–11 ms revealed retinal isomerization, the valine flip and the movement of the α-helix, which are the characteristic features occurring in the millisecond time range of the KR2 photocycle, as observed previously (Skopintsev *et al.*, 2020 ▸). Compared with the data measured at SwissFEL, most of the features are observed in both datasets, although the electron density map from the BioMAX data shows less-defined densities owing to limitations in dose and radiation damage when collecting synchrotron data at RT (Leonarski *et al.*, 2023 ▸). Following the success of this experiment, the PSI decided to loan MicroMAX the JUNGFRAU 9M detector and continue collaborating during the SLS upgrade dark period.

## 8. Outlook

MAX IV laboratory offers a variety of synchrotron beamlines and sample preparation laboratories relevant to life sciences. Regular meetings are held across all beamlines supporting life sciences research to update one another on new developments and to explore synergies. The capabilities at MAX IV span a vast range of methodologies that can explore biological systems at different length scales. This includes small- and wide-angle X-ray scattering at CoSAXS, tomographic imaging at ForMAX, X-ray spectroscopy at BALDER, as well as spectromicroscopy and coherent imaging at SoftiMAX and NanoMAX. Moreover, a dedicated Biolab can support on-site experiments. Through regular discussions and joint roadshows, we aim to guide current and future users to the most suitable endstations. Most recently, a Glacios CryoEM has been installed at MAX IV. Although it is organizationally separate, belonging to Lund University and the Swedish national infrastructure for life sciences, SciLifeLab, it is situated next to MicroMAX, sharing laboratory space and resources, thus completing resources for the structural biology community.

The landscape of structural biology has shifted significantly with recent advances in cryoEM and computational methods such as *AlphaFold* and *RosettaFold*, raising questions about the future role of MX. Though MX is no longer the sole method for atomic-resolution structure determination, this view overlooks the vast scale of unresolved biological questions that cannot be answered by a single method. Instead, it necessitates the optimal use and combination of diverse structural techniques to fully understand the molecular basis of life. MX remains an essential tool in this effort, with one of its key advantages being its unmatched throughput. BioMAX and its associated auto-processing pipelines can collect and process hundreds of datasets per day, often requiring minimal resources to reach final structures. This is particularly useful for iterative structure-based drug development projects, where rapid turnover of protein–ligand data is critical. Additionally, MicroMAX enables the study of protein dynamics on the microsecond scale, providing insight into structural changes that occur during these dynamic processes. While MX may not dominate every application, its contributions remain vital and complementary to other techniques. A major challenge moving forward will be adapting to a growing user base of non-experts. Traditionally, synchrotron radiation facilities have operated under a model where expert users conduct and analyse experiments independently. To accommodate a broader range of users, this paradigm must evolve to offer greater support throughout the experimental process, from sample preparation to data analysis. FragMAX, our crystallographic fragment-screening platform, will serve as a key test case for opening MAX IV’s MX capabilities to new communities, including medicinal chemists, computational biologists and clinicians. Although fragment screening is largely a logistical challenge, the process of X-ray data collection and structure refinement is well suited for automation. Collaborations with facilities like LP3 and other platforms ensure that external users have access to the necessary upstream expertise, facilitating greater participation from non-experts. On the downstream side, fragment elaboration – moving from a screening hit to a lead compound – presents its own challenges. Advances in computational chemistry, such as the ability to screen vast virtual chemical spaces, will be crucial in this process. While MAX IV does not intend to host a fragment elaboration service, we are in discussions with experts at SciLifeLab and computational chemists at CBCS to explore how such workflows might be integrated into our operations. Although these capabilities are already in place, our future goal is to establish and support a new operational model that integrates all these infrastructures. Ultimately, our aim is to make BioMAX and MicroMAX accessible to a new generation of scientists, enabling them to focus on their research questions rather than the methods they must use. This approach ensures that MAX IV Laboratory will continue to play a significant role in advancing life sciences through MX.
